# Congenital Infantile Fibrosarcoma Causing Intestinal Perforation in a Newborn

**DOI:** 10.1155/2017/2969473

**Published:** 2017-06-12

**Authors:** Margarita Kaiser, Bernadette Liegl-Atzwanger, Eszter Nagy, Daniela Sperl, Georg Singer, Holger Till

**Affiliations:** ^1^Department of Paediatric and Adolescent Surgery, Medical University of Graz, Graz, Austria; ^2^Institute of Pathology, Medical University of Graz, Graz, Austria; ^3^Division of Paediatric Radiology, Medical University of Graz, Graz, Austria; ^4^Division of Pediatric Hemato-Oncology, Medical University of Graz, Graz, Austria

## Abstract

Congenital infantile fibrosarcoma (CIF) is a rare malignant mesenchymal tumor and only 14 cases have been reported with gastrointestinal manifestation. We report about a female newborn delivered per emergency cesarean section at 34 weeks of gestation. Postnatally, she rapidly developed an acute abdomen and sonographic evidence of intestinal perforation requiring laparotomy on the first day of life. A perforated 2 × 3 cm sized spherical tumorous structure of the jejunum was identified. Due to unknown histopathology at this point and unclear resectional margins, she received a temporary ileostomy, which was closed two months later. Histopathology revealed a congenital intestinal fibrosarcoma without the characteristic ETV6-NTRK3 fusion transcript. In conclusion, this rare tumor must be considered as differential diagnosis of intestinal perforations in newborns.

## 1. Introduction

Congenital infantile fibrosarcoma (CIF) represents a malignant mesenchymal tumor accounting for 10–20% of soft-tissue tumors diagnosed in the early years of life [1]. CIF is a nonrhabdomyosarcoma derived from mesenchymal cells and is composed of malignant fibroblasts within a collagen background [1, 2]. Most cases share the distinctive feature of the specific translocation t(12;15) leading to the gene fusion ETV6-NTRK3 [1, 3].

Approximately 40% of CIFs are diagnosed in infants younger than 3 months or even prenatally. Nearly 80% of the cases are diagnosed within the first year of life [1]. A predominant occurrence in male patients has been observed [2].

The extremities, head, neck, and trunk represent the most frequent sites of occurrence. The ovaries, mesentery, and the retroperitoneum, however, are seldom involved [1]. Only a few cases of CIF located in the gastrointestinal tract have been described in the currently available literature [2].

Herein, we report a rare case of congenital infantile fibrosarcoma of the intestine without the classical ETV6-NTRK3 fusion.

## 2. Case Report

**Figure 1:**
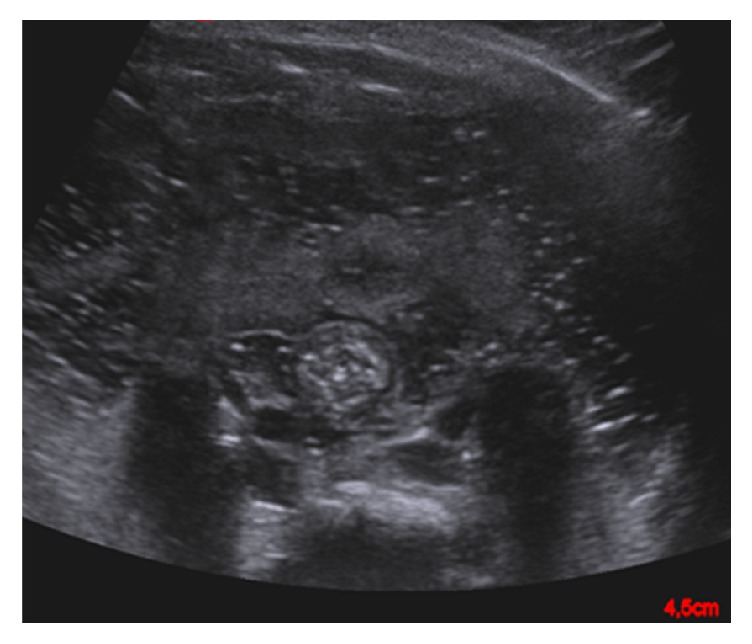
Ultrasound performed on the first day of life revealed free intraabdominal fluid.

The female patient was born per emergency cesarean section at 34 weeks of gestation because a volvulus was suspected on prenatal ultrasound. The birth weight was 2,270 g. Postnatally, the APGAR score was 8/8/9. The clinical condition, however, rapidly declined and the newborn developed tachypnea and tachycardia. An abdominal ultrasound revealed free fluid as a sign of meconium perforation (Figure 1). Both a volvulus and malrotation could be excluded.

**Figure 2:**
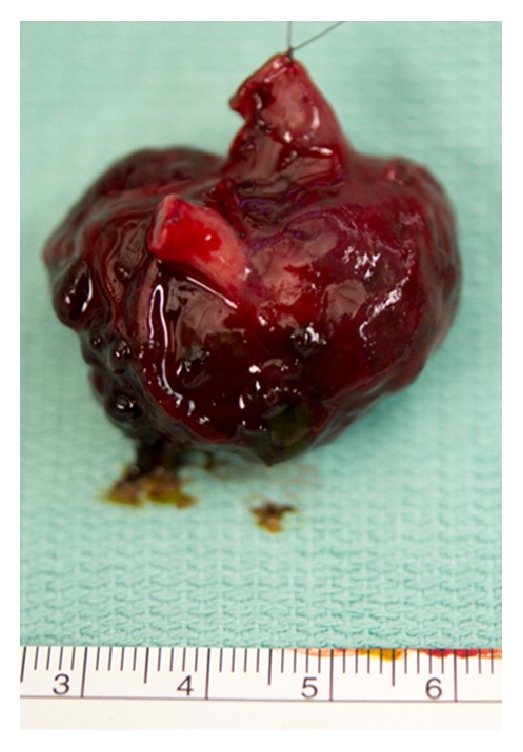
Intraoperative photograph showing the tumorous spherical structure (3 × 2 cm) of the distal jejunum.

The newborn was immediately taken to the operating room and underwent laparotomy. Intraoperatively, a perforated 2 × 3 cm sized spherical tumorous structure of the jejunum could be detected (Figure 2). No pathologic lymph nodes were seen during the operation. The whole intestinal segment including the tumorous structure was resected and the bowel was exteriorized with a double barrel jejunostomy due to unknown histopathology or clear resectional margins at this time.

**Figure 3:**
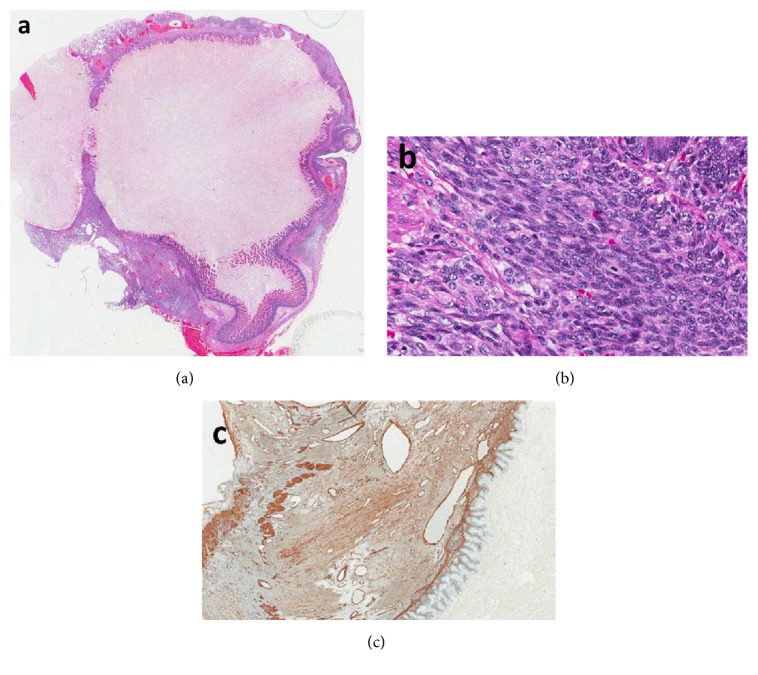
Histopathology revealed circumferential spindle cell proliferation involving mainly the submucosa and muscularis propria (a); the tumor consisted of spindle cells with tapering nuclei and indistinctive eosinophilic cytoplasm (b); immunohistochemistry showed focal positivity of tumor cells for SMA (c).

Histopathological examination confirmed complete removal of the tumor. HE stains revealed a circumferential cellular mesenchymal spindle cell proliferation involving mainly the submucosa and muscularis propria (Figure 3(a)). The tumor consisted of spindle cells with tapering nuclei and indistinctive eosinophilic cytoplasm (Figure 3(b)). The tumor cells were arranged in long intersecting fascicles. Up to 7 mitoses per high power field could be detected. Immunohistochemistry revealed focal positivity of tumor cells for SMA (Figure 3(c)). All other performed immunohistochemical stains (CK, AE1/AE3, Cam5.2, EMA, CD34, Desmin, CD116, and ß-catenin) were negative. FISH analysis demonstrated the lack of ETV-6 gene rearrangement. This finding was confirmed by performing the Archer Fusion Plex sarcoma Panel.

The further postoperative course was uneventful. The baby developed well and constantly gained weight. Therefore, the jejunostomy was closed at an age of two months. An MRI scan in the third month of life was unsuspicious and the patient was free of symptoms.

## 3. Discussion

The present case report describes a female newborn suffering from intestinal perforation due to a congenital infantile fibrosarcoma located in the jejunum. Complete removal was performed on the first day of life.

The exact etiology of CIF still remains unclear. However, a cytogenetic abnormality consisting of translocation t(12;15)(p13:q25) bearing the fusion of Tel gene EVT6 with TrkC gene has been shown for CIF [4, 5]. For the intestinal form of CIF, cases with and without this translocation have been reported [1, 3]. Some case presentations have not specifically examined this cytogenetic abnormality [6]. CIF most frequently affects the extremities, trunk, head, and neck [2, 3]. The occurrence in the gastrointestinal tract is confined to case reports and only 14 cases have been described in the literature [3, 6]. Nine of these cases involved the small bowel (*n* = 6 ileum, *n* = 2 jejunum, and *n* = 1 duodenum) and in 5 patients the colon was affected [3]. One of the two cases with occurrence in the jejunum lacked the ETV6-NTRK3 fusion transcript; in the other report, gene analysis has not been performed [3, 7]. The present report therefore represents the second patient with a jejunal manifestation of CIF without the abovementioned cytogenetic abnormality.

Approximately 40% of CIFs are diagnosed in infants younger than 3 months of age [1]. Moreover, nearly all cases located in the gastrointestinal tract are found within the first days of life [3]. The clinical presentation is acute in most cases and includes perforation and intestinal occlusion like in our patient. Recently, Zeytun and coworkers have presented a case with CIF of the ileocecal region that was detected incidentally without symptoms [6]. Preoperatively, the authors have performed magnetic resonance imaging (MRI) revealing a lesion in the right lower quadrant with hypointense contrast uptake on T1-weighted images and hyperintense contrast uptake on T2-weighted images. However, extensive imaging has not been performed in most of the reported cases due to the acute presentation necessitating immediate operative exploration [3].

In most cases of intestinal CIFs explorative laparotomy with subsequent resection of the tumor and end-to-end anastomosis was performed [2, 6]. Due to an unclear intraoperative diagnosis and the emergency situation in a newborn child, we have decided to create an jejunostomy instead of an end-to-end anastomosis. Recently, Scirè et al. have described a case of a laparoscopically managed ileal infantile fibrosarcoma in a female newborn examined for intestinal occlusion and suspected intussusception [1].

In contrast to adult fibrosarcomas, the infantile form is considered as a low-grade malignant tumor with an excellent prognosis [1, 2]. For the intestinal occurrence complete surgical resection has been shown to be curative and if the margins of resection are disease-free there is no need for additional chemo- or radiotherapy [1]. Neoadjuvant chemotherapy only plays a role in cases where surgery has limited value because operative resection would affect vital structures or would have mutilating consequences [8]. However, recurrences have not been reported in patients with intestinal CIFs [1–3, 6, 9–15].

In conclusion, the present report adds a patient with a jejunal CIF without ETV6-NTRK3 fusion transcript to the literature. In cases of intestinal perforation in a newborn, intestinal CIF can be included as a rare but possible differential diagnosis.

## References

[1] Scirè G., Mantovani A., Zampieri N. (2014). Transumbilical laparoscopic treatment of Congenital Infantile Fibrosarcoma of the Ileum. *La Pediatria Medica e Chirurgica*.

[2] Kim H. Y., Cho Y. H., Byun S. Y., Park K. H. (2013). A case of congenital infantile fibrosarcoma of sigmoid colon manifesting as pneumoperitoneum in a newborn. *Journal of Korean Medical Science*.

[3] Berrebi D., Fournet J.-C., Boman F. (2015). Intestinal congenital/infantile fibrosarcoma: a new clinico-pathological entity?. *Pediatric Surgery International*.

[4] Dubus P., Coindre J. M., Groppi A. (2001). The detection of Tel-TrKC chimeric transcripts is more specific than TrkC immunoreactivity for the diagnosis of congenital fibrosarcoma. *Journal of Pathology*.

[5] Knezevich S. R., McFadden D. E., Tao W., Lim J. F., Sorensen P. H. B. (1998). A novel ETV6-NTRK3 gene fusion in congenital fibrosarcoma. *Nature Genetics*.

[6] Zeytun H., Okur M. H., Basuguy E. (2016). Congenital-infantile fibrosarcoma of the ileocecal region: the first case presentation. *Pediatric Surgery International*.

[7] Shima Y., Ikegami E., Takechi N. (2003). Congenital fibrosarcoma of the jejunum in a premature infant with meconium peritonitis. *European Journal of Pediatric Surgery*.

[8] Parida L., Fernandez-Pineda I., Uffman J. K. (2013). Clinical management of infantile fibrosarcoma: A retrospective single-institution review. *Pediatric Surgery International*.

[9] Parmar V., Peters R. T., Cheesman E., Edi-Osagie N., Craigie R. J. (2014). Congenital infantile fibrosarcoma of the colon: a case series and literature review. *Pediatric Surgery International*.

[10] Islam S., Soldes O. S., Ruiz R., Geiger J. D. (2008). Primary colonic congenital infantile fibrosarcoma presenting as meconium peritonitis. *Pediatric Surgery International*.

[11] van Niekerk M. L., Nel W. A., Slavik T. (2010). Infantile fibrosarcoma of the ileum presenting with congenital bowel obstruction. *Journal of Pediatric Surgery*.

[12] Buccoliero A. M., Castiglione F., Degl'Innocenti D. R. (2008). Congenital/infantile fibrosarcoma of the colon: Morphologic, immunohistochemical, molecular, and ultrastructural features of a relatively rare tumor in an extraordinary localization. *Journal of Pediatric Hematology/Oncology*.

[13] Shearburn E. W., Teja K., Botero L. M., Shaw A. (1975). Pancreaticoduodenectomy in the treatment of congenital fibrosarcoma of the duodenum. *Journal of Pediatric Surgery*.

[14] Rizkalla H., Wildgrove H., Quinn F., Capra M., O'Sullivan M. J. (2011). Congenital fibrosarcoma of the ileum: Case report with molecular confirmation and literature review. *Fetal and Pediatric Pathology*.

[15] Bruno C., Caliari G., Zampieri N., Segala D., Pozzi-Mucelli R. (2014). Congenital fibrosarcoma of the bowel: Sonographic description of a rare case of neonatal intestinal obstruction. *Journal of Clinical Ultrasound*.

